# Histo-Molecular Intratumoral Heterogeneity in Meningiomas: A Narrative Review

**DOI:** 10.3390/cancers18081206

**Published:** 2026-04-10

**Authors:** Nourou Dine Adeniran Bankole, Tuan Le Van, Luc Kerherve, Edouard Morlaix, Jean-François Bellus, Kerima Belhajali, Julian Lopez, Pierre De Buck, Alia Sayda Houidi, Walid Farah, Maxime Lleu, Olivier Baland, Cathy Cao, Ahmed El Cadhi, Jacques Beaurain, Thiebaud Picart, Moncef Berhouma

**Affiliations:** 1 Neurosurgery Department, Dijon University Hospital Center, 21000 Dijon, France; 2 INSERM, Imaging Brain & Neuropsychiatry iBrain U1253, Université de Tours, 37032 Tours, France; 3 Neurosurgery Department, Hospices Cilvils de Lyon-Pierre Wertheimer Hospital, 69500 Bron, France; 4 INSERM 1052 UMR 5286, Research Cancerology Center of Lyon, 69008 Lyon, France; 5 Functional and Molecular Imaging Team, Centre National de la Recherche Scientifique 6302, Molecular Chemistry Institute (ICMUB), University of Bourgogne, 21000 Dijon, France

**Keywords:** intratumoral heterogeneity, meningioma, histology, molecular profiling

## Abstract

**Background**: Meningiomas, the most common primary intracranial tumors, are predominantly benign, but high-grade variants show marked aggressiveness, histo-molecular heterogeneity, and treatment resistance. Although the 2021 WHO CNS classification integrates molecular and histopathologic criteria, substantial inter- and intratumoral variability still limits prognostic accuracy and treatment effectiveness. The goal was to provide insight regarding the histo-molecular intratumoral heterogeneity (ITH) of meningioma and examine its clinical implications. **Methods**: A narrative review was performed in accordance with PRISMA guidelines. PubMed and Google Scholar were screened for studies on “meningioma” and “intratumoral heterogeneity” published up to 28 July 2025. Eligible studies included original human research reporting histological or molecular heterogeneity with clinical relevance. **Results**: Eighteen studies comprising 2952 meningioma patients (mean age 59.4 ± 14.8 years, range 16–85) were included. Integrated cytogenetic, molecular, and spatial analyses, including FISH, karyotyping, scRNA-seq, CNV profiling, and spatial transcriptomics, revealed multilayered histo-molecular heterogeneity. Histologically, regional variations in morphology and proliferative index increased with tumor grade. Genomic diversity, marked by recurrent losses of 1p, 14q, and 22q and transcriptionally distinct subclones, defined a complex tumor architecture. Spatial and temporal analyses demonstrated subclonal expansion, stepwise clonal evolution, and therapy resistance, particularly in recurrent tumors. Functionally, SULT1E1^+^ subclones and COL6A3-mediated macrophage–tumor interactions emerged as potential key drivers of malignancy, recurrence, and radioresistance. **Conclusions**: Histo-molecular diversity underlies meningioma progression, recurrence, and therapeutic resistance. Standardization of ITH assessment, integration of AI-based spatial analytics, and the development of subclone-specific therapies are essential next steps toward advancing precision neuro-oncology.

## Simple Summary

Meningiomas are the most common benign brain tumors, but some aggressive forms behave unpredictably and are difficult to treat. This variability is partly explained by intratumoral heterogeneity, meaning that different regions of the same tumor can have distinct histological and molecular characteristics. In this review of 18 studies including 2952 meningioma patients, we found that meningiomas exhibit complex and multilayered heterogeneity at genetic, cellular, and spatial levels, which contributes to tumor progression, recurrence, and resistance to therapy. Specific subpopulations of tumor cells and interactions with the tumor microenvironment may play a key role in these processes. Understanding this diversity is essential to improving diagnosis, predicting outcomes more accurately, and developing more personalized treatment strategies.

## 1. Introduction

Meningiomas are the most common primary intracranial tumors, accounting for more than one-third of central nervous system neoplasms, with a lifetime incidence of ~1% [1,2,3]. While the majority are low-grade and clinically indolent, high-grade meningiomas demonstrate marked biological heterogeneity, aggressive behavior, and high recurrence rates despite surgery and radiotherapy [4,5,6].

Systemic therapies remain ineffective, and the role of intratumoral heterogeneity (ITH) and tumor evolution under treatment is still poorly defined [1]. Traditionally, prognosis has been guided by histopathologic grade and extent of resection, but emerging molecular insights have refined risk stratification [7]. The 2021 WHO Classification of CNS tumors integrates molecular features, with TERT promoter mutations and CDKN2A/B deletions as criteria for grade 3 designation [8,9,10]. Genomic patterns also vary by tumor location: convexity and spinal meningiomas frequently harbor 22q loss and NF2 mutations, whereas skull base tumors are enriched for AKT1, TRAF7, SMO, and PIK3CA alterations, highlighting the clinical relevance of molecular profiling [1,11].

Despite these advances, histologic grade and recurrent genetic drivers alone do not fully capture clinical behavior. Growing evidence indicates that ITH spanning genetic, epigenetic, transcriptional, and functional levels plays a central role in tumor progression, therapeutic resistance, and recurrence [12,13,14,15]. This complexity not only confounds current diagnostic and prognostic frameworks but also limits the effectiveness of targeted therapies. Therefore, a more comprehensive understanding of the spatial, temporal, and molecular dimensions of ITH is essential to improve risk stratification, enhance prognostic accuracy, and guide the development of more effective therapeutic strategies.

In this study, the aim was to systematically characterize histo-molecular ITH in meningiomas using integrated genomic, spatial, and functional approaches, with the goal of elucidating mechanisms of tumor progression and informing precision diagnostic and therapeutic strategies for high-grade meningioma.

## 2. Narrative Review Search Methods

The methodology for this narrative review followed a systematic approach to the literature search, study selection, data extraction, and synthesis. We conducted a comprehensive search on PubMed and Google Scholar using Boolean operators combining the keywords “Heterogeneity,” “Intratumoral”, “Histo-molecular,” and “Meningioma”. The search was limited to studies in English or French published up to 28 July 2025, with detailed queries provided in the Supplemental appendix Queries. Titles, abstracts, and full texts were screened to identify studies focusing on histo-molecular ITH in meningioma. Inclusion criteria comprised studies that reported histo-molecular ITH in meningioma and their clinical relevance. Exclusion criteria included case reports, review articles, and studies involving non-human meningioma.

**Figure 1:**
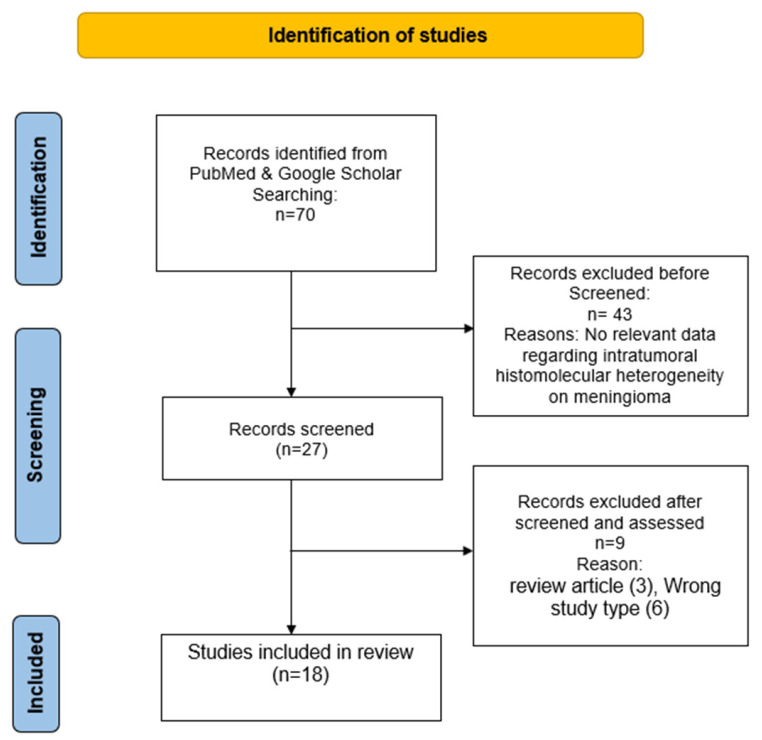
Flowchart for study selection.

The search initially identified 70 studies. Of these, 43 (61.5%) were excluded at the title/abstract screening stage, and 9 (12.8%) were excluded after full-text review. A total of 18 studies (25.7%) met the inclusion criteria and were retained, encompassing 2952 meningioma samples (59.4 ± 14.8, range 16–85 Years) (Supplementary Table S1; Figure 1).

Data extraction was performed using a standardized form, capturing study design, sample size, the type of ITH assessment methodology, histological features, type of molecular heterogeneity, and clinical relevance. Given the narrative nature of this review, no formal assessment of study quality or risk of bias was undertaken. Data were synthesized and reported narratively, focusing on histo-molecular profile, genetic profile, patterns of heterogeneity, and observed correlations between heterogeneity and clinical outcomes (see Table S1). Also, pooled statistics were calculated using measures of central tendency and dispersion.

## 3. Methodologies Used to Assess ITH

ITH in meningiomas has been characterized through a multimodal analytical framework integrating cytogenetic, molecular, spatial (by comparing samples from repeat surgeries), and functional approaches (Table S1). Classical cytogenetic methods, including karyotyping, comparative genomic hybridization (CGH), and interphase or multicolor fluorescence in situ hybridization (FISH/iFISH), have identified recurrent chromosomal aberrations such as losses of 1p, 14q, and 22q, and revealed clonal diversity across tumor grades [5,16]. Flow cytometry and immunohistochemical analyses enable rapid, quantitative assessment of proliferative heterogeneity through the evaluation of DNA content, detection of aneuploidy, and measurement of cellular proliferation indices [17,18,19,20].

Recent advances in single-cell RNA sequencing (scRNA-seq) and copy number variation (CNV) analyses have enabled the identification of transcriptional and genomic subclones with distinct proliferative, metabolic, and immune-interacting phenotypes [11,20,21,22]. Spatial transcriptomics and imaging mass cytometry further delineate regional variations in gene expression, proliferation, and immune–vascular architecture, capturing microregional heterogeneity within the tumor microenvironment [20,21]. Complementary platforms such as patient-derived meningioma organoids reproduce native tumor complexity, allowing mechanistic investigation of subclonal behavior [22], while oncogenetic tree modeling reconstructs clonal evolution and predicts recurrence risk based on genetic progression patterns [5,20]. Collectively, these integrative methodologies provide a comprehensive understanding of meningioma heterogeneity and histo-molecular assessment.

## 4. Types of Intratumoral Histo-Molecular Heterogeneity

### 4.1. Histological and Spatial Heterogeneity

**Figure 2:**
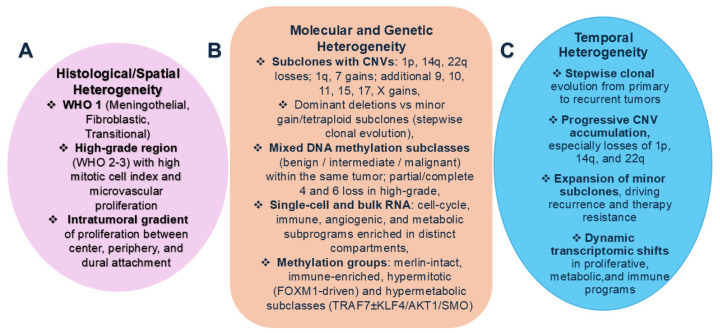
Histomolecular heterogeneity in meningiomas. (**A**) Histological–spatial heterogeneity with mixed WHO grade 1 patterns, focal high-grade regions, and intratumoral proliferation gradients between center, periphery, and dural attachment. (**B**) Molecular and genetic heterogeneity with recurrent CNVs, mixed methylation subclasses, and distinct transcriptional programs (cell-cycle, immune, angiogenic, and metabolic). (**C**) Temporal heterogeneity with stepwise clonal evolution from primary to recurrent tumors, progressive CNV accumulation, and expansion of aggressive subclones.

Across studies, histological and spatial heterogeneity in meningiomas are tightly interconnected, jointly reflecting regional differences in morphology, proliferation, and molecular architecture within the same tumor (Table S1, Figure 2). Low-grade (WHO 1) tumors often show regional architectural differences or mixed histological patterns, such as meningothelial, fibroblastic, and transitional, while higher-grade (WHO 2–3) meningiomas display pronounced microregional variability in cellularity, mitotic rate, necrosis, and vascularisation [23,24,25]. Intraoperative flow cytometry identified significant intratumoral spatial heterogeneity in proliferative indices across the tumor center, periphery, and dural attachment. The highest proliferative fractions localized to the dural attachment in low-grade, slow-growing tumors, whereas higher-grade lesions showed increased proliferation within central and peripheral regions associated with pial vascular supply [17]. Moreover, subclonal chromosomal and transcriptomic diversity mirrors histologic complexity: in high-grade meningiomas, regions with rhabdoid morphology frequently coincide with high-mitotic zones and mosaic alterations in p16, H3K27me3, and Ki-67 expression, indicating that these aggressive phenotypes tend to colocalize within the same spatially heterogeneous niches rather than occurring uniformly throughout the tumor [23,26,27]. Spatial heterogeneity in meningiomas reflects regional differences in cellular composition, proliferation, and molecular architecture within the same tumor [9,17,20,21]. Spatial profiling further reveals that these histologically distinct niches correspond to underlying subclonal chromosomal and transcriptomic diversity, including compartmentalized deletions such as 1p and 22q and localized enrichment of immune, angiogenic, and metabolic programs. High-grade tumors, in particular, show architectural compartmentalization in which highly proliferative, immune-interacting, and angiogenic niches coexist with relatively quiescent regions within the same mass [20,21,28]. These findings underscore that histological heterogeneity in meningiomas extends beyond grade classification, reflecting the interplay between regional morphology, proliferative dynamics, and underlying molecular diversity. The coexistence of distinct histologic patterns within single tumors, particularly in higher grades, suggests that meningioma progression involves localized microenvironmental influences and subclonal evolution. Spatial variation in proliferation indices further supports the presence of functionally distinct tumor compartments, which may contribute to uneven growth, recurrence risk, and treatment resistance.

### 4.2. Molecular and Genetic Heterogeneity

Meningiomas exhibit extensive molecular and genetic heterogeneity encompassing chromosomal, mutational, epigenetic, and transcriptional alterations that vary by tumor grade and subtype [5,9,12,16,20,21,22,29,30] (Table S1). Classical cytogenetic studies revealed frequent chromosomal losses of 1p, 14q, and 22q and gains of 1q, 7, 9, 10, 11, 15, 17, and X, defining key subclonal populations and reflecting stepwise clonal evolution [5,9,16,20,21]. Single-cell and FISH analyses identified both dominant clones with large-scale chromosomal deletions and minor subclones with chromosomal gains or tetraploidy, indicating progressive diversification during tumor evolution [16,20,21]. High-grade meningiomas show uniform and complex karyotypic abnormalities, whereas low-grade tumors may display focal or cryptic changes [9,16]. Single-cell and multi-omics analyses have further delineated transcriptional heterogeneity, revealing distinct subclones enriched for cell-cycle, immune, angiogenic, and metabolic pathways [20,21,22,26,28]. DNA methylation profiling demonstrates intratumoral epigenetic heterogeneity, with the coexistence of benign, intermediate, and malignant methylation subclasses within a single tumor. High-grade tumors are further characterized by recurrent partial or complete copy number losses involving chromosomes 4 and 6 [31]. Molecular outliers, such as SULT1E1^+^ subpopulations, influence macrophage polarization and contribute to malignant behavior [22], whereas MDM2 amplification and TERT promoter mutations arise heterogeneously during tumor progression or recurrence [12,29]. Methylation analysis further defines merlin-intact, immune-enriched, and hypermitotic groups, with hypermitotic splitting into proliferative (FOXM1-driven and worst prognosis) and hypermetabolic (metabolism-driven) subgroups that refine risk prediction [18,19]. Complementary, genomics and proteomics reveal NF2/chr22-loss in about half of high-grade tumors vs. non-NF2 skull base benign grade tumors subtypes (TRAF7 ± KLF4/AKT1/SMO). Within NF2 tumors, ANXA3 emerges as a targetable proliferative driver [11,18,19]. Therefore, molecular and genetic analyses reveal that meningiomas are highly heterogeneous tumors characterized by diverse chromosomal, epigenetic, and transcriptional alterations. Recurrent CNVs and the coexistence of multiple subclonal populations highlight a stepwise clonal evolution from low- to high-grade disease. Single-cell and methylation studies further uncover functionally distinct subpopulations driving proliferation, immune modulation, and angiogenesis. The emergence of molecular outliers, such as SULT1E1^+^ and TERTp-mutant clones, underscores the dynamic and adaptive nature of tumor evolution, reinforcing the need for integrated, multi-omics approaches to better predict behavior and guide targeted therapy.

### 4.3. Temporal Heterogeneity

Temporal heterogeneity in meningiomas arises from clonal evolution and progressive genetic diversification over time, particularly between primary and recurrent tumors [12,20,21,29] (Table S1). Longitudinal cytogenetic and single-cell sequencing studies demonstrate that meningiomas acquire additional chromosomal losses and gains, most commonly involving 1p, 14q, and 22q, alongside the emergence of new subclones during recurrence or malignant transformation [21,29]. Even minor genetic subclones at initial presentation can expand and dominate subsequent tumor stages, driving recurrence, therapeutic resistance, and malignant progression [12,21]. Transcriptomic analyses further reveal dynamic temporal shifts in proliferative, metabolic, and immune-interacting programs, reflecting adaptive remodeling of the tumor microenvironment during recurrence [20,21]. Collectively, these findings indicate that meningiomas evolve through a stepwise, time-dependent accumulation of molecular alterations, underscoring the importance of longitudinal genomic monitoring to predict recurrence, refine risk stratification, and guide targeted therapeutic strategies.

## 5. Clinical Implications

Across studies, a link has been established between the ITH and WHO grade, prognosis, and therapeutic response in meningiomas. Low-grade (WHO grade 1) meningiomas generally exhibit limited or regional heterogeneity, whereas high-grade (WHO grade 2–3) tumors display extensive genetic, molecular, and spatial complexity. This increased heterogeneity correlates with more aggressive clinical behavior, earlier recurrence, and reduced responsiveness to conventional treatments [9,16,20,21,30,32]. Homogeneous and multi-chromosomal aberrations, particularly deletions in 1p, 14q, and 22q, are strongly associated with poor prognosis and higher recurrence rates, while tumors lacking major chromosomal alterations tend to grow more slowly and recur less frequently [5,9,16,23,30].

Temporal and clonal evolution contribute to malignant transformation, emphasizing the prognostic importance of detecting minor but genetically advanced subclones that can drive recurrence and treatment resistance [12,21]. Functionally, distinct proliferative and immune-interacting subpopulations such as SULT1E1^+^ cells promote tumor aggressiveness, radioresistance, and macrophage polarization, yet also represent promising therapeutic targets [22,23]. Collectively, current evidence highlights a paradigm shift toward molecularly integrated grading and personalized management, where mapping ITH refines prognostic accuracy, guides risk stratification, and informs the development of precision therapies in meningioma care. Several targeted therapies show promise in recurrent high-grade disease, including everolimus plus octreotide and sunitinib, while combination strategies such as alpelisib with trametinib highlight the potential of co-targeting signaling pathways [33,34,35,36]. Tumor growth rate reduction may serve as a sensitive endpoint to evaluate treatment efficacy. However, despite these advances, the management of high-grade meningiomas remains challenging and requires further validation of emerging therapies.

## 6. Future Directions and Perspectives

Despite significant advances in defining histo-molecular diversity, standardized frameworks for assessing ITH in meningiomas are still lacking. Current diagnostic workflows, which often rely on single-biopsy histopathology and bulk molecular analyses, fail to capture the spatial and temporal complexity of these tumors. Tumor models, particularly patient-derived organoids, are emerging as valuable platforms to recapitulate intratumoral heterogeneity. Multiregional and single-cell sequencing studies have consistently demonstrated that histologically homogeneous meningiomas can nonetheless harbor substantial intratumoral heterogeneity, characterized by the coexistence of genetically and transcriptionally distinct clonal populations as well as microregional variability in cellular composition and signaling states. These findings highlight the limitations of conventional histopathological assessment in fully capturing tumor complexity. In this context, the development and adoption of standardized methodological frameworks for multiregional sampling, spatially resolved molecular profiling, and robust quantification of clonal architecture are likely to be critical. Such standardization would enable a more accurate and reproducible characterization of tumor biology, improve the prediction of recurrence risk and clinical outcomes, and enhance comparability across studies and institutions. Moreover, the implementation of these approaches would support the incorporation of intratumoral heterogeneity metrics into future WHO classification systems and advance their integration into precision oncology strategies, ultimately facilitating more personalized and biologically informed therapeutic decision-making.

Furthermore, the integration of artificial intelligence (AI) and spatial transcriptomics represents a transformative opportunity for ITH characterization. AI-driven image analytics and machine learning algorithms can detect subtle histopathological and molecular correlates of heterogeneity that are underestimated by conventional microscopy. Meanwhile, spatial omics approaches, including multiplex imaging and proteogenomic mapping, enable simultaneous visualization of gene expression, chromosomal aberrations, and immune–vascular networks within intact tumor sections [21,28]. These multimodal datasets can be computationally modeled to reconstruct evolutionary trajectories, identify resistant subregions, and inform surgical margin assessment. Ultimately, integrating AI-based morpho-molecular analytics with spatial omics also has the potential to refine tumor classification and guide adaptive, patient-specific therapeutic strategies.

Therapeutically, elucidating ITH provides a foundation for targeted and combined treatment strategies in high-grade and recurrent meningiomas. The identification of SULT1E1^+^ initiating clones [22] and COL6A3-mediated macrophage–tumor interactions [21] underscores the potential for selective targeting of malignant and immune-regulatory subpopulations to suppress progression and recurrence. Moreover, the heterogeneity of TERTp mutations, MDM2 amplification, and DNA methylation subtypes highlights the need for adaptive molecular therapies that address dynamic clonal evolution. Future clinical trials should incorporate ITH-informed biomarkers for patient stratification and personalized therapy, integrating small-molecule inhibitors, immune modulators, and radiosensitizers such as SRT1720 [21,22,29]. The convergence of real-time ITH profiling, AI-based predictive modeling, and molecularly guided therapy promises to significantly advance precision neuro-oncology for refractory and high-grade meningiomas [20,21,22,29].

## 7. Limitations of the Review

This narrative review is limited by several factors. First, there was substantial heterogeneity in study design, sample size, and methodology across the included studies, along with missing age data in 10 of the 18 studies. These introduce variability that limits direct quantitative comparisons. Second, most available studies focus on small, regionally sampled cohorts, often lacking standardized protocols for multiregional or longitudinal assessment of ITH. Third, data integration across molecular, spatial, and clinical domains remains incomplete, with limited validation in prospective or therapeutic contexts. Despite these limitations, this review summarizes the most comprehensive, up-to-date evidence on histo-molecular heterogeneity and its clinical implications in meningioma biology.

## 8. Conclusions

Intratumoral histo-molecular heterogeneity represents a defining feature of meningioma biology, shaping tumor evolution, recurrence risk, and therapeutic response. Multimodal approaches, including single-cell and spatial transcriptomics, advanced cytogenetics, and functional assays (intraoperative flow cytometry, DNA ploidy and proliferation index analysis, immunohistochemical proliferation markers, and patient-derived organoid models with drug and radiosensitivity testing), have revealed that meningiomas evolve through stepwise clonal diversification, with genetic, epigenetic, and immune-driven mechanisms underpinning malignant progression. High-grade tumors exhibit spatially compartmentalized subclones and temporal genomic evolution that challenge conventional grading and necessitate molecularly integrated diagnostics. Standardized ITH assessment, AI-assisted spatial analytics, and subclone-targeted therapeutics hold promise for translating biological complexity into precision care. Future research should aim to validate these integrative approaches in longitudinal clinical settings, fostering the transition toward personalized neuro-oncology in meningioma management.

## Data Availability

Not appliable.

## Supplementary Materials

The following supporting information can be downloaded at: https://www.mdpi.com/article/10.3390/cancers18081206/s1, Table S1: Summary of relevant findings from included study.
