# Supplementary material for: Histo-Molecular Intratumoral Heterogeneity in Meningiomas: A Narrative Review
Source: Cancers (Basel). 2026 Apr 10;18(8):1206. doi: 10.3390/cancers18081206 (PMC13114662; doi:10.3390/cancers18081206)
Supplement: Supplementary file 1 [file cancers-18-01206-s001.zip › Queries.pdf]

28/07/2025

Search: **Intratumoral Heterogeneity in Meningioma**
("intratumor"[All Fields] OR "intratumoral"[All Fields] OR "intratumorally"[All Fields] OR "intratumorous"[All Fields] OR "intratumour"[All Fields] OR "intratumoural"[All Fields] OR "intratumourally"[All Fields]) AND ("heterogeneic"[All Fields] OR "heterogeneities"[All Fields] OR "heterogeneity"[All Fields] OR "heterogeneous"[All Fields] OR "heterogeneously"[All Fields] OR "heterogenic"[All Fields] OR "heterogenicity"[All Fields] OR "heterogenities"[All Fields] OR "heterogenity"[All Fields] OR "heterogenization"[All Fields] OR "heterogenized"[All Fields] OR "heterogenizing"[All Fields] OR "heterogenous"[All Fields] OR "heterogenously"[All Fields]) AND ("meningioma"[MeSH Terms] OR "meningioma"[All Fields] OR "meningiomas"[All Fields])

28/07/2025

Search: **Histomolecular heterogeneity of meningiomas**
"Histomolecular"[All Fields] AND ("heterogeneic"[All Fields] OR "heterogeneities"[All Fields] OR "heterogeneity"[All Fields] OR "heterogeneous"[All Fields] OR "heterogeneously"[All Fields] OR "heterogenic"[All Fields] OR "heterogenicity"[All Fields] OR "heterogenities"[All Fields] OR "heterogenity"[All Fields] OR "heterogenization"[All Fields] OR "heterogenized"[All Fields] OR "heterogenizing"[All Fields] OR "heterogenous"[All Fields] OR "heterogenously"[All Fields]) AND ("meningioma"[MeSH Terms] OR "meningioma"[All Fields] OR "meningiomas"[All Fields])
