# Supplementary material for: Histo-Molecular Intratumoral Heterogeneity in Meningiomas: A Narrative Review
Source: Cancers (Basel). 2026 Apr 10;18(8):1206. doi: 10.3390/cancers18081206 (PMC13114662; doi:10.3390/cancers18081206)
Supplement: Supplementary file 1 [file cancers-18-01206-s001.zip › Table S1.pdf]

Table S1: Summary of relevant findings from included study

Author (Year)	Study design Sample size (N) and number of meningiomas (n)	Age years Mean (SD or IQR) Females(F)= n	Methods used to assess ITH	Histology heterogeneity Types	Molecular/genetic heterogeneity types	Clinical relevance
Scheck et al[9] (2009) USA	Retrospective study n=77 meningiomas	NA	Regional multizonal fluorescent in situ hybridization (FISH) for chromosomal abnormalities (1p, 14q, 22q) across multiple tumor regions.<br><br>Affymetrix-based gene expression microarrays.<br><br>Immunohistochemistry (MIB-1 Ki-67 for proliferation index).<br><br>Pooled review of cytogenetics, karyotyping, CGH, LOH, Sanger sequencing.	Substantial regional heterogeneity: chromosomal and molecular aberrations often confined to specific zones within the tumor, especially in grade I, with grade II/III showing more widespread (homogeneous) changes.	Deletions and gains within 1p, 14q, and 22q, some grade I tumors display no visible abnormalities, suggesting cryptic or focal genetic changes, Grade I tumors often display regional or subclonal alterations or none at all, whereas grade II and III tumors consistently harbor uniform and complex abnormalities, most commonly deletions in 1p, 14q, and 22q, with trisomy 22 occasionally observed in grade III.	Intratumoral genetic heterogeneity (in grade I) corresponds to less aggressive behavior and delayed or absent recurrence, especially after subtotal resection.<br><br>Homogeneous high-grade or multifocal genetic alterations (especially in 1p, 14q, 22q) are statistically linked to higher grade, more aggressive growth, and early recurrence.<br><br>Morphology and grade alone insufficient for predicting outcome combination of proliferation index (MIB-1) and regional molecular analysis improves risk stratification.
Huang et al[22] (2023) China	Experimental, translational research n=21 meningiomas	NA	Single-cell RNA sequencing (scRNA-Seq)	WHO Grade 1<br>WHO Grade 2<br>WHO Grade 3	Unique SULT1E1⁺ cell subpopulation acting as an initiating and regulatory clone ; Functional heterogeneity mediated through macrophage polarization (M2-type) and tumor-immune interactions.	The discovery of the SULT1E1⁺ subpopulation as a driver of malignancy and recurrence provides a potential therapeutic target, with SRT1720 identified as a promising agent for systemic treatment and radiosensitization in refractory high-grade meningiomas.
Oya et al [17] (2019) Japan	Prospective study N=50 patients n= 50 meningiomas	64.0 (35-85) F= 32 (64%)	Intraoperative rapid flow cytometry (iFC): performed on fresh tumor samples to quantify proliferative ability.<br><br>Proliferation Index (PI): defined as the ratio of aneuploid cells (with abnormal chromosome number) to total cells.<br><br>Comparative analysis: correlation of PI with MIB-1 labeling index (LI), annual growth rate (AGR), and intratumoral distribution patterns.	WHO Grade 1<br>WHO Grade 2<br>WHO Grade 3	PI varied across tumor regions (center, periphery, dural attachment), reflecting microregional histologic diversity.<br><br>aneuploidy measurement (chromosomal number abnormalities) representing genomic-level heterogeneity within tumor subregions ;	significant intratumoral heterogeneity in proliferative potential, correlating with tumor growth rate and vascular features (development of pial feeders). iFC provides a rapid, intraoperative alternative to MIB-1 LI for assessing tumor aggressiveness.

Sayague´s et al (2002)[16] Spain	Observational cytogenetic analysis N=70 meningiomas patients	56 ± 15 (16–81) ; F= 42(69%)	Interphase Fluorescence In Situ Hybridization (FISH) applied to detect numerical chromosomal abnormalities in chromosomes 1, 9, 10, 11, 14, 15, 17, 22, X, and Y	WHO Grade 1<br>WHO Grade 2<br>WHO Grade 3	Most frequent losses: Chromosome 22 (53%), 14 (19%), X (23% males), Y (32% males).<br><br>Frequent gains: Chromosomes 1, 9, 10, 11, 14, 15, 17, X.<br><br>Clonal diversity:<br><br>Losses → involved large cell populations (major clones).<br><br>Gains → limited to small subclones/tetraploid cells.<br><br>Distinct association patterns between chromosomal gains and losses (e.g., monosomy 22 with monosomy X in females; monosomy 14 with loss of Y in males).	-Meningiomas are genetically heterogeneous, comprising multiple chromosomal subclones with distinct progression pathways.<br><br>-Chromosome 22 loss is a potential key early event, while chromosome gains may represent later clonal evolution.<br><br>-The prognostic value of detecting clonal diversity, as specific chromosomal alterations and their distribution within the tumor may correlate with tumor aggressiveness, recurrence risk, and progression potential.
URBSCHAT et al[5] (2011) Germany	Retrospective study N=661 meningiomas patients	57.3 (12.8) ; F= 482 (72.9%)	-Classical karyotyping to identify chromosomal aberrations.<br><br>-Single-cell cytogenetic analysis to detect subclonal chromosomal differences within individual tumors.<br><br>-Oncogenetic tree modeling to reconstruct the order of genetic events and compute a genetic progression score (GPS) predictive of recurrence.	WHO Grade 1<br>WHO Grade 2<br>WHO Grade 3	Primary aberrations : Normal karyotype or monosomy 22 (common in low-grade tumors).<br><br>Secondary aberrations : Additional losses of autosomes and sex chromosomes (common in anaplastic tumors).<br><br>Intratumoral heterogeneity: Detected in 33.4% of cases (224/661), showing distinct cytogenetic clones within single tumors.<br><br>Advanced subclones (with multiple accumulated losses) were identified as drivers of progression and recurrence.<br><br>Single-cell level diversity highlights that even one genetically advanced clone can determine clinical behavior.	intratumoral cytogenetic heterogeneity is a key determinant of meningioma recurrence and progression.<br><br>Genetic progression score (GPS) and identification of advanced subclones provide more accurate recurrence prediction than bulk tumor analysis.<br><br>Emphasizes the need to assess subclonal structure rather than relying solely on the dominant cytogenetic pattern, improving prognostic precision and potential treatment stratification.

Sayague´ s et al [29] (2004) Spain	Observational cytogenetic study N=125 meningiomas patients	58 ± 15 (16 to 82) ; F= 81 ; 64.8%	Multicolor interphase fluorescence in situ hybridization (iFISH) for 11 chromosomes and flow cytometry for DNA content and aneuploidy detection; comparative analysis between both methods to map clonal architecture and DNA instability.	WHO Grade 1 (benign), WHO Grade II (atypical), and WHO Grade 3 (anaplastic). Single-clone tumors corresponded to Grade 1; multi-clonal tumors (≥2 clones) were observed in higher grades (2–3), reflecting progression-associated histological diversity.	- Chromosome losses: 22q (most frequent early event), X, Y, and 1p deletions.<br>- Chromosome gains : observed in a minority (4%) of early clones, often associated with tetraploidization (31%) during evolution.<br>- Distinct clonal evolution pathways between benign and atypical/anaplastic tumors, suggesting independent molecular origins rather than linear progression.	- Intratumoral clonal diversity was present in about 55% of cases and strongly correlated with tumor grade and aggressiveness.<br>- Benign (Grade I) tumors: often single-clone, with isolated 22q loss.<br>- Atypical/anaplastic (Grade II-III) tumors : multi-clonal, complex chromosomal alterations, and aneuploidy linked to aggressive behavior and recurrence.<br>- Distinct evolutionary pathways imply that high-grade meningiomas may not always arise from benign precursors, influencing prognostic assessment and therapeutic targeting.

Lucas et al[23] (2024) USA	Retrospective, observational study<br>N=502 meningiomas	NA	Histological and molecular grading by 2021 WHO CNS tumor classification.<br><br>Immunohistochemistry for Ki-67, H3K27me3, and p16.<br><br>Spatial transcriptomic profiling using 10x Genomics Visium Spatial assay (on FFPE tissue).<br><br>Spatial protein profiling (NanoString Digital Spatial Profiler, multiplexed antibody panels).<br><br>Multiplexed sequential immunofluorescence microscopy (seqIF, COMET platform).<br><br>Single-cell RNA sequencing and spatial deconvolution.<br><br>Bulk targeted DNA and RNA sequencing.<br><br>Copy number variant (CNV) analysis by inferCNV.<br><br>CRISPR interference (CRISPRi) in patient-derived and cell line co-culture models.<br><br>Pharmacological screening in 3D co-cultures	High-grade (WHO grade 2/3) meningiomas exhibit regionally distinct histology, focal areas of :<br><br>-Increased cellularity<br><br>-High mitotic count sufficient for WHO grade 3 in subregions<br><br>-Rhabdoid morphology (BAP1 mutation linked)<br><br>-Mosaic changes in p16, H3K27me3, and Ki-67 within spatially distinct regions.	Intratumor heterogeneity at the genomic and transcriptomic levels seen in :<br><br>-Oncogenic driver mutations (e.g., BAP1 inactivation, CDKN2AB homozygous deletion, TERT promoter mutations, ARID1A, ASXL1, MN1)<br><br>-Divergent sub-clonal CNVs: losses and gains of chromosomes or arms (e.g., 1p, 4q, 9p, 10q, 14q, 18, 19q, 22q, 1q gain)<br><br>-Distinct DNA methylation groups within same tumor<br><br>-Spatially divergent transcriptional clusters with variable proliferation, differentiation, ECM remodeling, immune signatures, and cell cycle activation<br><br>-Diverse patterns of PI3K-AKT and MAPK signaling, immune infiltration.	Current WHO/histological or molecular classification systems can group genetically and phenotypically diverse tumors together, limiting prognostic or therapeutic stratification.<br><br>-High-grade meningioma recurrence and treatment resistance are linked to spatial expansion of subclonal CNVs, decreased immune infiltration, decreased MAPK, increased PI3K-AKT pathway activity, and increased proliferation.<br><br>-Findings underscore the need for spatial and molecular profiling of recurrent tissue (not only archival) for clinical decisions.<br><br>-Preclinical modeling shows that combination therapies rather than monotherapies are required to target heterogeneous tumor clones (CDK4/6 inhibitors for CDKN2AB loss; HDAC or PI3K-AKT pathway inhibitors for epigenetic regulator loss; combination recommended).

Goutagny et al [24] (2012) France	Retrospective observational cohort study N=119 n=287 meningiomas	NA	Histological review and WHO 2007 grading of all resected meningiomas.<br><br>Immunohistochemistry for progesterone receptor expression.<br><br>Genomic characterization by high-density single nucleotide polymorphism (SNP) array to study copy number variations and chromosome instability.<br><br>NF2 gene mutation analysis by direct sequencing and gene dosage testing.	Each specimen often showed a predominant histological subtype along with up to six additional recognizable subtypes :<br>Main subtypes identified were transitional, fibroblastic, meningothelial, papillary, psammomatous, microcystic, angiomatous, and cartilaginous metaplasia.<br>WHO Grade 1<br>WHO Grade 2<br>WHO Grade 3	Frequent chromosome arm losses, increasing with histological grade.<br><br>Most common losses: 22q, 1p, 18q, 6p.<br><br>Chromosome instability was higher in grade II/III tumors (mean losses per grade 1 : 2.2, 2 : 5.7, 3 : 4.5).<br><br>Diversity of chromosomal gains, losses, and loss of heterozygosity within and between tumors in same patient.<br>Unique breaks on 22q indicative of independent clonal origins in multicentric cases.	Most NF2-associated meningiomas demonstrated minimal growth and were indolent, suggesting that proactive treatment is not always necessary.<br><br>De novo meningiomas were rare (8.7%) but exhibited higher growth rates and more aggressive behavior, requiring closer monitoring and more aggressive treatment.<br><br>Meningiomas with brain edema tended to be larger, grow faster, and were more often higher grade.<br><br>Grades II/III represented only 4.2% of meningiomas in this NF2 cohort, not more aggressive than sporadic cases.

Peyre et al[12] (2017) France	Multicenter, retrospective cohort study. N=57 meningiomas patients	60 years	Centralized pathological review using the 2016 WHO Classification of Brain Tumors.<br><br>Analysis of mitotic index and presence of frank histological anaplasia.<br><br>Assessment and discrimination between de novo and secondary anaplastic cases.<br><br>TERT promoter mutation analysis via direct sequencing.<br><br>TERT promoter methylation analysis (by methylation-specific PCR on bisulfite-converted DNA).	WHO grade 3(anaplastic) meningioma defined by:<br><br>High mitotic index (≥20 mitoses per 10 high-power fields) and/or overt anaplasia (loss of meningothelial differentiation, pseudo-carcinomatous/-sarcomatous features).<br><br>De novo anaplastic meningiomas: Could present with "histological anaplasia alone" (without high mitotic count) or with both anaplasia and increased mitoses.<br><br>Secondary anaplastic meningiomas: All were diagnosed as grade III based on an increased mitotic index that progressively increased over relapses, reaching ≥20 mitoses per 10 HPF.<br><br>Heterogeneity also in the histological presentation between de novo and secondary cases.	TERT promoter mutation analysis: Overall, TERT promoter mutations found in 14% of anaplastic meningiomas (8/57).<br><br>Both C228T and C250T variants detected; not restricted to either de novo or secondary groups.<br><br>TERT mutation status assessed through consecutive tumor samples in some secondary cases.<br><br>TERT promoter methylation: Analysis performed in 33 anaplastic meningiomas; 91% positive.<br><br>Variability in acquisition of TERT promoter mutations (found in pre-anaplastic and at progression samples).	De novo anaplastic meningiomas had longer overall survival (OS) than secondary anaplastic meningiomas.<br><br>De novo median OS: 3.1 years; Secondary: 2.1 years (p=0.02).<br><br>Within de novo tumors, those diagnosed on histological anaplasia alone (not just high mitotic count) had a markedly better prognosis (median OS : 6.2 years vs. 2.6 years).<br><br>In secondary cases, time to first relapse as a lower-grade meningioma was the strongest predictor of OS.<br><br>Gross total resection (GTR) improved prognosis in de novo cases.<br><br>TERT promoter mutation not correlated with OS overall, but in secondary anaplastic subgroup, TERT mutation was associated with shorter recurrence-free survival.<br>Lack of TERT promoter methylation (very rare) may be associated with better prognosis.

Juratli et al,[28] (2017) USA	Retrospective, multi-institutional N=26 N=64 meningiomas	55.6 ± 16.6 F= 11 (42.3%)	-Sanger sequencing and fluorescence PCR for TERT promoter mutations (C250T, C228T).<br><br>-Matched blood DNA to confirm somatic status.<br><br>-Sequencing of ATRX and DAXX for alternative telomere pathway mutations.<br><br>-Anchored multiplex PCR (Archer FusionPlex) for TERT rearrangements/fusions.<br><br>-Next-generation sequencing (SNaPshot) for other cancer gene mutations.<br><br>-Immunohistochemistry for ATRX protein expression.<br><br>-Longitudinal analysis (matched primary and recurrent samples; spatial ITH by multi-site sampling in recurrent tumors).	WHO grade 1 to 3	-TERTp mutation status was heterogeneous within a single tumor (found at certain but not all sites in the same patient/tumor).<br><br>-TERTp mutations typically absent at initial diagnosis but acquired during malignant progression or at recurrence.<br>-In post-radiation meningiomas, TERTp mutations were uncommon.	Acquisition of TERTp mutations during tumor progression is associated with poor prognosis and significantly shorter overall survival (median 2.7 vs 10.8 years for TERTp-mutant vs wildtype, p=0.003).<br><br>TERTp mutation is independently associated with poor outcome in multivariate analysis.<br><br>Marked spatial heterogeneity may cause underdiagnosis of aggressive subclones if only one region is sampled, supporting tumor evolution model with late-arising, aggressive TERTp-mutant clones.
Vega et al, (2022)[30] Sweden	Retrospective study, N=12	NA	Genome-wide DNA methylation profiling (Illumina Infinium EPIC BeadChip array).<br><br>Chromosomal copy number alteration (CNA) analysis inferred from methylation array data.<br><br>Histopathological evaluation (including WHO grade).<br><br>Tumor purity estimation (multiple computational algorithms and pathology).<br><br>Immunohistochemistry (including MDM2 protein detection for amplification validation).	WHO grade 1 to 3	MDM2 proto-oncogene amplification (rare, linked to malignant progression) was heterogeneous in a high-grade meningioma.<br><br>DNA methylation subtype heterogeneity was detected in three high-grade meningiomas and one low-grade meningioma, with co-existence of benign, intermediate, and malignant methylation subtypes or subclasses within the same tumor.<br>Copy number heterogeneity: partial or whole loss of chromosomes 4 and 6 was observed within three high-grade meningiomas.<br><br>High numbers of differentially methylated CpG sites (DMPs) found, especially in high-grade tumors	Intratumor heterogeneity of DNA methylation and CNAs poses risks for misdiagnosis and sampling bias, potentially affecting WHO grading and biomarker interpretation.<br><br>Particularly for maglinant progression markers like MDM2 amplification, heterogeneity may impact patient stratification and selection for targeted therapies.<br><br>Higher heterogeneity (methylation/DMPs, CNAs) associated with high-grade meningiomas, indicating greater risk of recurrence and poor prognosis; methylation-based subclassification improves prediction over classic WHO grading.

Che et al[27] (2024) China	Experimental / Bioinformatics study N=8	NA	Single-cell RNA sequencing (scRNA-seq) and bioinformatics pipelines for clustering and differential gene expression.<br><br>Gene Ontology (GO), KEGG pathway, and Gene Set Enrichment Analysis (GSEA).<br><br>Protein-protein interaction (PPI) network mapping.<br><br>Copy number variation (CNV) analysis at single-cell resolution using inferCNV.<br><br>Functional enrichment and spatial mapping of cell populations.	Identified six MGC (meningioma cell) subtypes, including ECM-remodeling, cycling S, cycling G2/M, SSTR2-positive, and NOS-MGCs. ECM-remodeling MGCs localized mainly at the brain–tumor interface, showing distinct transcriptional and spatial features.	CNV analysis identified distinct chromosomal alterations by subtype: ECM-remodeling cells (with losses in chromosomes 1, 16, 22), cycling subpopulations (gains in chromosomes 2, 12, 15, 20; losses in 13, 14).<br><br>ECM remodeling cells exhibited lower CNV scores, suggesting a lower degree of malignancy compared to other tumor subtypes.<br><br>Key molecular regulators (FN1, CTNNB1, IL6, JUN, FOS, MYC) identified as central in ECM-remodeling cell networks.	ECM-remodeling MGCs exhibit low malignant potential, strong adhesion, and stable interaction with the basement membrane, possibly limiting invasion and recurrence.<br><br>Cycling MGCs (S and G2/M phases) show higher CNV load and malignancy, potentially linked to recurrence and progression.<br><br>CNV patterns (loss of chr 22, 1, 16) consistent with classical meningioma drivers.<br><br>Suggests molecular profiling at the single-cell level provides superior insights for assessing tumor aggressiveness and recurrence risk beyond WHO grade.
Wang et al (2022)[25] USA	Experimental, single-cell transcriptomic and multi-omics study N=8	NA	Single-cell RNA sequencing (scRNA-seq) for both immune and non-immune cell profiling.<br><br>Copy number variant (CNV) analysis at single-cell level using CONICSmat.<br><br>T cell receptor (TCR) sequencing for clonotype diversity.<br><br>Imaging mass cytometry and immunohistochemistry for protein marker validation and cell spatial positioning.<br><br>Unsupervised clustering, differential gene expression, and gene ontology enrichment analyses.	Evidence of heterogeneity seen in cellular subtypes and microenvironmental composition at the single-cell transcriptomic level rather than classic histomorphology.<br><br>Varying proportions of immune cell (lymphoid, myeloid) subtypes and functionally distinct non-immune (endothelial, mural, and fibroblast) populations, implying substantial underlying cellular and microenvironmental diversity.	Clear subclonal CNV heterogeneity at the single-cell level within individual tumors, confirmed by detection of distinct chromosomal aberrations (e.g., deletions in 14q, 16q, 19q, 22q; amplifications in 1q, 5p, 6p, 7p/q, 8q, 9p/q, 11p, 15q, 20q).<br><br>Multiple discrete tumor subpopulations ("clonal groups") with unique CNV and transcriptomic profiles were identified in the same tumor, frequently mapping to specific biological pathways (e.g., metabolism, cell division, ECM organization).<br><br>CNV subclones associated with distinct gene expression and functional pathway enrichment; some clonal groups enriched for division, others for metabolic and stress responses.	Subclonal heterogeneity in meningiomas, as revealed by single-cell CNV and transcriptional mapping, may underpin variable clinical behavior, prognosis, and therapy response.<br><br>Shared T cell receptor clonotypes between adjacent dura and tumor suggest local immune surveillance, but tumor-infiltrating T cells are generally less expanded and exhibit an exhausted phenotype compared to those in dura, indicating a tumor-modified immune milieu.<br><br>The diversity of both immune and non-immune cell populations within and between tumors underscores the necessity for spatially resolved, multi-modal sampling to capture clinically actionable heterogeneity.

Magill et al[31]<br>(2020)<br>USA	Prospective multiplatform genomics, epigenetics, transcriptomics, histopathology, and radiomics study<br>N=13	65 (52-79) ;	Bulk RNA sequencing and bioinformatics (gene expression)<br><br>DNA methylation profiling (Illumina EPIC array)<br><br>Copy number variant (CNV) analysis inferred from methylation data<br><br>Tumor phylogenetic trees (clonal evolution tracking)<br><br>Quantitative MR imaging (Apparent Diffusion Coefficient mapping, perfusion)<br><br>Histology (H&E), immunofluorescence for Ki-67, FOXM1<br><br>Development and use of a meningioma-cerebral organoid model (live imaging, single-cell RNA-seq, CRISPRi, pharmacology with CDH2 inhibitor)	Spatially-defined samples revealed regional variation in cellularity, collagen deposition, necrosis, and proliferation (Ki-67 and FOXM1), notably more pronounced in higher-grade tumors<br><br>WHO grade was stable for each tumor but heterogeneous proliferative and cellular characteristics were found within tumors, especially grades 2-3	Uniform CNV profiles across spatial sites in grade I tumors; marked CNV heterogeneity (chromosomes 1p, 14q, 22q losses; 11q, 17q gains) in high-grade tumors<br><br>Transcriptomic heterogeneity (RNA-seq) and DNA methylation heterogeneity were prominent in grades 2-3, with enrichment for developmental, immune, and proliferation gene sets (FOXM1 pathway, Wnt signaling, neuronal development)<br><br>Regions with high ADC on MR imaging corresponded to distinct molecular programs (CDH2, PTPRZ1 gene expression)<br><br>Single-cell and spatial mapping revealed clonal evolution and compartmentalized genomic rearrangements	Regions with greater genomic instability, high proliferation, and specific transcriptomic programs (FOXM1, CDH2, PTPRZ1) are linked to tumor recurrence, resistance, and poor clinical outcomes<br><br>DNA methylation profiling is robust for prognosis but is vulnerable to sampling bias; multiple spatial samples better capture heterogeneity<br><br>Preoperative MR imaging (ADC mapping) identifies aggressive, molecularly distinct regions—suggesting image-guided targeted sampling improves prognostic accuracy and therapy stratification<br><br>CDH2 inhibition (ADH-1) blocks meningioma proliferation and tumorigenesis in vitro/organoid models, indicating new molecular targets for therapy
Lee et al[21]<br>(2025)<br>Republic of Korea	Longitudinal Experimental study<br>N=7 patiens<br>n = 14 meningiomas	NA	Droplet-based 10x Genomics snRNA-seq for high-resolution transcriptomes and gene expression profiling.<br><br>Copy number variation (CNV) analysis by inferCNV at single-cell resolution.<br><br>Differential gene expression and pathway enrichment between primary and recurrent tumors.<br><br>RNA velocity and latent time analysis to model cellular state transitions and evolution.<br><br>Immunohistochemistry and cell type marker validation for spatial distribution.<br><br>Functional validation by siRNA-mediated knockdown in meningioma cell lines.	Both primary and recurrent meningiomas consisted of diverse cellular compositions and hierarchies.<br><br>Tumor cells mapped into four main molecular subtypes (MG1-immunogenic, MG2-NF2 wild-type, MG3-hypermetabolic, MG4-proliferative).<br><br>Recurrent samples showed enrichment of hypermetabolic and proliferative cells.	High intra- and inter-tumoral diversity in CNVs, especially losses of ARID1A and NF2; amplification of angiogenesis genes ROBO1 and ROBO2 was frequent in recurrence.<br><br>Transcriptomic heterogeneity driven by activation of cell cycle (FOXM1), DNA repair (POLQ, BRIP1), and extracellular matrix genes (COL6A3).<br><br>COL6A3 emerged as the major marker of risk and recurrence overexpressed in recurrent and high-grade meningiomas and predicts poor outcome and relapse-free survival.	Recurrent meningiomas are characterized by cell cycle activation, proliferative dynamics, and immunosuppressive microenvironments (high infiltration of C1Q and M2 macrophages).<br><br>COL6A3 was validated as a risk gene and therapeutic target; its knockdown reduced proliferation and cell cycle activity in vitro.<br><br>High COL6A3 expression correlates with worse survival in large validation cohorts.<br><br>Single-cell resolution mapping and subtype analysis provide mechanistic insight into recurrence and therapy resistance, supporting multi-targeted approaches for high-risk meningiomas.

Choudhury et al[18]<br>2023<br>USA	Retrospective, multi-institutional molecular reanalysis<br>n=565 meningiomas	NA	Genome-wide DNA methylation profiling<br><br>Copy number variant (CNV) inference from methylation data	WHO grade 1-3 Hypermetabolic and Proliferative subgroups were enriched in WHO grade 3 tumors within the Hypermitotic group	Merlin-intact: Enriched for NF2 expression and relatively fewer NF2-deleting CNVs<br><br>Immune-enriched: Enriched in immune cell signatures on methylation deconvolution, fewer HLA-deleting CNVs, and hypomethylation/overexpression of meningeal lymphatic genes<br>Hypermitotic: High Ki-67, high genomic instability, frequent CDKN2A/B loss and USF1 gain, and enrichment of FOXM1 target genes overall<br><br>Strong enrichment of FOXM1 target genes (MKI67, CCNB1, CDC20, etc.).<br><br>Differential burden and pattern of CNVs across groups and subgroups (NF2 loss, CDKN2A/B deletion, HLA locus alterations).	Concordance and discordance with other schemes (gene-expression types, methylation families, integrated grade/score) reveal that the same tumor can occupy different risk strata depending on the model, reflecting "informatic heterogeneity" on top of biological heterogeneity.<br><br>The demonstration that Hypermitotic meningiomas split into Proliferative vs Hypermetabolic subgroups shows that even within a single high-risk class, there is meaningful molecular heterogeneity with distinct biology and outcomes.
Shah et al[19]<br>2025<br>UK	Retrospective analysis of meningioma tumor samples from UK biobanks<br>n=118 meningiomas	NA	Targeted NGS for driver mutations (NF2, AKT1, KLF4, TRAF7) and co-occurrences (maftools); variant annotation (ANNOVAR, CGI, FATHMM-MKL); oncogenicity (oncoEnrichR)<br><br>Label-free LC-MS/MS proteomics (MaxQuant, Perseus) for differential protein expression/clustering; validation by Western blot, Simple Wes, PRM-MS<br><br>DNA methylation profiling (Illumina EPIC) for NF2 subgroups<br>Functional assays: Lentiviral shRNA knockdown (ANXA3), EdU proliferation, Westerns (MCM2, p-ERK1/2), orthotopic xenografts (bioluminescence/GFP)	WHO grade 1-3 tumors analyzed, but focus on grade 1,<br>NF2 grade 1 tumors split into two proteomic/histologic subgroups: Cluster 1 (fibrous/transitional) ; Cluster 2 (psammomatous/mixed),	NF2 (56%, mostly frameshift/truncating, severity score 3); AKT1^{E17K}/Q79K (12%); KLF4^{K409Q} (11%); TRAF7 (16%), Co-occurrences<br>TRAF7 with AKT1 or KLF4 ;<br><br>ATM-BIVM-ERCC5 (DNA repair); PIK3C2B-SDHD (PI3K/metabolism); ERCC4-MET (DNA repair/growth factor).<br>NF2 mutually exclusive with non-NF2 drivers ;<br>AKT1^{E17K}/TRAF7: Upregulated oxidative phosphorylation, CLIC3/CRABP2/GMDS/Pyruvate carboxylase.<br><br>KLF4^K409Q/TRAF7: Downregulated signaling; upregulated CD44/Endoglin/E-cadherin/Anion exchange protein 2.<br><br>NF2$^{mut/-}$: Two proteomic clusters with distinct mutations (Cluster 1: SMARCB1 loss, DNA damage/p53 functions, int-A/ben-1 methylation; Cluster 2 : β-catenin/nuclear receptor functions, ben-1/ben-3 methylation); upregulated ANXA3 (Annexin-3).	The study reveals heterogeneity within grade 1 meningiomas (co-mutations, proteomic clusters), explaining aggressive behavior despite low grade.<br><br>NF2 subgroups (proteomic/genetic) show distinct molecular functions (DNA repair vs. β-catenin), histotypes, and methylation classes, suggesting further stratification beyond WHO grade.<br><br>ANXA3 as a therapeutic target: Upregulated specifically in NF2 tumors (all grades); shRNA knockdown reduces proliferation (EdU/MCM2/p-ERK), abolishes growth in grade 3 xenografts-proposing personalized therapy for the most common meningioma driver (NF2, ~56%).

Clark et al[11]<br>2013<br>USA	Retrospective genomic discovery study<br>N =300 meningiomas	NA	Chromosome 22 copy-number analysis<br>Targeted Sanger/NGS resequencing of top candidates (NF2, TRAF7, KLF4, AKT1, SMO).	WHO grade I (benign, vast majority), grade II (atypical).<br><br>Histologic subtypes: secretory (all TRAF7+KLF4^{K409Q}), meningothelial (often AKT1^{E17K})	NF2/chr22 loss (~50%, 149 tumors):<br>NF2 coding mutations (n=108) ± chr22 loss; high genomic instability (multiple large-scale CNVs); higher grade (2).<br>Non-NF2 mutant (~25–30%): TRAF7 mut (~24%, n=72).<br><br>KLF4^{K409Q} (zinc finger mutation, always with TRAF7).<br><br>AKT1^{E17K} (PI3K activator, often with TRAF7).<br>SMO mut (~5%, n=11): Recurrent L412F/W535L (Hedgehog activation)	Meningioma molecular subtypes correlate with location, behavior, and therapy. NF2/22q-loss tumors arise in hemispheric or lateral skull base regions, show genomic instability, higher grade, and recurrence risk, and rely mainly on surgery and radiotherapy. Non-NF2 tumors (TRAF7, KLF4, AKT1, SMO) occur in the medial/anterior skull base, are usually grade 1, chromosomally stable, and have excellent prognosis. These profiles enable targeted therapies (e.g., PI3K or Hedgehog inhibitors), support subtype-guided trials, and help avoid overtreatment of indolent tumors.

Footnotes : NA (not available),
